# Clozapine Response for Vocal Tics in Schizophrenic Patients: A Case Report With Literature Review

**DOI:** 10.7759/cureus.14111

**Published:** 2021-03-25

**Authors:** Gulshan Begum, Stanley Nkemjika, Olaniyi Olayinka, Tolu Olupona, Ayodeji Jolayemi

**Affiliations:** 1 Psychiatry, Interfaith Medical Center, Brooklyn, USA; 2 Public Health/Epidemiology, Georgia State University, Atlanta, USA; 3 Psychiatry and Behavioral Sciences, Interfaith Medical Center, Brooklyn, USA

**Keywords:** schizophrenia, vocal tics, risperidone, clozapine

## Abstract

Antipsychotics have been documented in the literature as the most effective pharmacological treatment for tics thus far. Additionally, evidence in the literature has shown that typical and atypical antipsychotics are effective for the treatment of tic disorders in patients who are diagnosed with schizophrenia and other psychiatric illnesses. This evidence is typified as atypical antipsychotic medications, such as risperidone, aripiprazole, and olanzapine, have been documented as being effective for motor tics, particularly in Tourette’s syndrome. Despite the level of evidence with regard to antipsychotics, there is no published literature on the role of clozapine-based treatment for persistent vocal tics in schizophrenia. We present a case of severe adult-onset persistent vocal tics in a schizophrenic patient who was admitted for acute psychotic exacerbation. The patient's vocal tics as well as his comorbid psychotic symptoms were not responsive to risperidone and paliperidone. The combination of risperidone with clozapine for psychosis showed improvements in his symptoms. In addition, the patient’s tics showed excellent response to risperidone and clozapine combination therapy. He was subsequently discharged to the community with clozapine 125 mg twice daily and paliperidone palmitate 156 mg every 28 days. The patient was psychiatrically stable without vocalization at the time of discharge. We suggest that clozapine augmentation therapy could be an approach in treatment-resistant vocal tics among schizophrenic patients who are refractory to atypical antipsychotics.

## Introduction

Vocal tics are common manifestations of Tourette's syndrome (TS), which is a rare neurobiological disease characterized by a combination of multiple motor tics for at least one year in duration [1]. TS is defined as sudden, rapid, recurrent, and non-rhythmic movements or vocalizations with varying degree of purposefulness and voluntariness [2]. Despite documented evidence of existing overlap in clinical symptoms and responsiveness to psycho-pharmacological treatment of tics, treatment of psychotic symptoms with comorbid tic symptom manifestation is rarely reported in the literature. Similarly, there is a dearth in the literature with regard to the choice of antipsychotic medications used in the management of tics in psychotic disorders as there are varied reports. The only evidence documented in the literature is the olanzapine-based vocal tic-related schizophrenia management [3]. Thus, we present a case of a chronic schizophrenic patient who manifested vocal tics in addition to psychotic symptoms and successfully treated with risperidone augmented with clozapine. Hence, this is probably the first reported case of vocal tics in an adult schizophrenic patient.

## Case presentation

Our patient was a 28-year-old Jewish Caucasian male who presented to the department with grossly disorganized speech, aggressive, screaming, yelling, and repetitive loud bizarre vocalization mimicking barking. On initial evaluation by a psychiatrist at our department, the patient was acutely psychotic, grandiose, paranoid delusions, aggressive, and extremely irritable. He was exhibiting irresistible urge for vocalization that mimicked barking and a sense of relief afterward.

The patient's first diagnosis of schizophrenia was nine years ago, and the reported duration of vocal tics was three months prior to presentation at our institution. His mental health problems started at the age of 19 years with poor social interaction and emotional dysregulation. His symptoms gradually progressed to poor impulse control, anger outbursts, physical violence, and cognitive difficulties. At the age of 19, he began to experience auditory hallucinations and disorganization of thought process (talking irrational, loose associations, and thought blocking) that led to the diagnosis of schizophrenia. Detail evaluation of history, a review of medical records, and collateral information from his mother revealed that he has had multiple hospitalizations and has undergone multiple antipsychotic trials since the onset of illness. His psychosis and affective symptoms were poorly managed by neuroleptics over the last eight years. The patient is experiencing tic-like symptoms in addition to his psychotic symptoms for the last three months. Per report from the patient's mother, his language, motor skill, and social skills development were normal during childhood.

Routine biochemical investigations including liver function tests, serum calcium, phosphorus, TSH (thyroid-stimulating hormone), T4, and electrolytes were normal. Urinalysis and hemogram were normal. Valproic acid levels were within the therapeutic level. Electrocardiogram (EKG) was normal. A computerized tomography (CT) scan of the brain was unremarkable. After a thorough medical workup, treatment was initiated with risperidone and valproic acid. On the fifth day, he was started on long-acting injectable antipsychotic paliperidone palmitate 234 mg IM followed by a second initiation dose of 156 mg. Due to lack of response with risperidone and a past history of unsuccessful treatment with various neuroleptics, clozapine titration protocol was initiated on the 14th day. After two weeks of clozapine augmentation, at a dose 125 mg twice a day the patient started showing improvement of his psychotic symptoms. It was observed that vocalizations also gradually improved as he underwent treatment with clozapine. Significant improvement was noted in the following weeks. He was stable psychiatrically without vocalization on the eighth week and discharged to the community. The patient was kept on Clozapine 125 mg twice a day schedule as his symptoms were in remission with this dose.

## Discussion

We described the case of an adult patient of chronic schizophrenia with frequent vocalization resembling vocal tics. Documented initial diagnostic consideration for new adult-onset tic-like sounds includes a number of distinct conditions that must be excluded. Additionally, the presence of vocal tics as a symptomatic manifestation of schizophrenia has been implied in previous literature [4], as there are limited clinical evidential reports on tics and tic-like episodes. The absence of any known baseline significant premorbid abnormalities in language, motor skill, or social development while growing up also makes this case report unique. Hence, this incidental finding may be a component of schizophrenia spectrum.

Though genetic factors have been hypothesized and described in the literature to play role in the pathogenesis of schizophrenia [5], there is limited evidence with regard to the presence of tics episode in such cases. Similarly, there are no or only a few reports on the genetic heritability of tic-like syndromes. It is noteworthy that the genetic-linked schizophrenia episodes usually manifest at childhood and get diagnosed as the very early onset schizophrenia [6]. Similarly, tic-like stereotypic behavior has been well documented in autistic spectrum disorder (ASD) [1], but the absence of ASD symptoms and no past history of ASD diagnosis in this patient makes this case unique. Additionally, the presence of no other organic pathology or previous psychiatric diagnosis in this patient’s medical history other than schizophrenia may be a focus for possible future research to understand its pathogenesis. Interestingly, the patient displayed occasional repetitive stereotyped grunting, throat-clearing vocalizations, and bark-like sounds as an isolated schizophrenia symptom, which is not documented as part of the criteria A of the DSM-5 (Diagnostic and Statistical Manual of Mental Disorders, 5th Edition) spectrum disorder other than identified disorganized behavior. Hence, this case report raises an issue worthy of inclusion as new symptomatology for schizophrenia spectrum disorder.

Pathophysiological research has identified dopaminergic hyperactivity pathways in tic disorders, which could also be attributed to the same mechanisms of schizophrenia [5]. The blocking effect of most antipsychotics such as aripiprazole, whose mechanisms is via partial dopamine and serotonin 2A antagonism at the presynaptic receptor, may lead to increase dopamine availability, thus inducing tics. This could be a probable link between schizophrenia and vocal tics manifestation in this case report, as this patient has a history of several antipsychotic trials in the past including aripiprazole. Despite this suggested unified neurophysiology of both tics (in this case vocal) and schizophrenic symptoms, this case is a rare presentation to the best of our understanding. Most studies have shown that adult-onset tics may be due to general medical condition such as stroke or ingestion of psychoactive compounds such as illicit drugs. Others have reported co-existence with comorbid psychological disorders such as obsessive-compulsive disorder [7], ASD, and attention-deficit hyperactivity disorder [8]. These considerations are undoubtedly very important in assessing and making diagnostic impressions in cases with similar presentation to our patient. It is noteworthy that our case lacked any concurrent or comorbid medical illness, no history of substance use, and no positive urine toxicology for any illicit substance use, which might explain his new symptoms. However, considering the chronicity of the patient’s schizophrenic disorder for more than nine years and his psycho-pharmacology history, antipsychotics-induced tics was strongly considered and ruled out.

Clozapine is an atypical antipsychotic drug with established superior efficacy for treatment-resistant schizophrenia with a low prevalence of extrapyramidal symptoms [9]. It has not been documented as the mainstay treatment for vocal tics or tic-like syndromes. Additionally, persistent vocal tics, other specified tic disorder, based on DSM-5 in patients with schizophrenia, and the responsiveness to antipsychotics have rarely been studied. On extensive literature search, we found limited evidence of tics symptom management in schizophrenics [3]. However, the most notable study identified is a documented case report of positive good response to olanzapine monotherapy [3]. Thus far, no other studies have replicated the use of atypical antipsychotics such as olanzapine as a treatment modality for schizophrenia-related tics. Rather, other conflicting studies have suggested that tic-like symptoms might manifest during treatment with atypical antipsychotics such as clozapine, quetiapine, atomoxetine, olanzapine, and aripiprazole [10]. Lindenmayer et al. reported that there were multiple cases of tic-like symptoms in patients on high-dose clozapine treatment regimen, which gradually resolved with reduction of the dosage strength [11]. On the other hand, a study suggested that atypical antipsychotics such as olanzapine were associated with the onset of tic-like symptom in chronic schizophrenic patients on medication [3].

The presence of vocal tics in a Jewish schizophrenic adult that was managed and resolved with clozapine administration is the vital point of intrigue to this case. Thus, there is a need for appropriate documentation of the unique findings, which will improve and bridge the gap in the literature. Additionally, this may spur further health-related research or clinical studies to replicate or refute the index findings in this case. This will aid in the understanding of treatment mechanism of such, cases which should be tailored to meet the needs of the patient and aim at maximum symptom reduction. Furthermore, systematic studies of these types of patients may help better understand the etiologic processes and the varied clinical presentations involved in schizophrenia. Though there are different suggestive management modalities for tic-like symptoms, such as reducing dosage or by adding valproate, clonazepam, or other antipsychotics, the use of clozapine in this patient was based on the resistant nature of the non-resolute acute symptoms of his presentation several weeks after inpatient management. The coincidental treatment and resolution of the vocal tic component of his mental disorder are unprecedented.

## Conclusions

In conclusion, given that our patient displayed disorganized or bizarre vocalizations and behaviors that were accompanied by negative symptoms such as amotivation, apathy, grandiose delusions, thought blocking, and characteristic course of marked decline in functioning, his symptoms responded at a lower dose clozapine along with risperidone. To our knowledge, this is the first case report of successful treatment with risperidone augmented with clozapine. This highlights the possibility that risperidone augmented with clozapine therapy may be considered as an ideal treatment option for schizophrenia patients who present with tic vocalization. Hence, the utilization of clozapine in the management of vocal tics for this patient is novel and the first reported case to the best of our knowledge.

## References

[1] Canitano R, Vivanti G (2007). Tics and Tourette syndrome in autism spectrum disorders. Autism.

[2] Robertson MM (2015). A personal 35 year perspective on Gilles de la Tourette syndrome: prevalence, phenomenology, comorbidities, and coexistent psychopathologies. Lancet Psychiatry.

[3] Cheng WJ, Liu HC, Huang MC (2007). Olanzapine monotherapy for late-onset vocal tics in a schizophrenic patient. Psychiatry Clin Neurosci.

[4] Telgote SA, Pendharkar SS, Kelkar AD, Bhojane S (2017). Very early-onset schizophrenia with secondary onset tic disorder. Indian J Psychol Med.

[5] Seeman P, Kapur S (2000). Schizophrenia: more dopamine, more D2 receptors. Proc Natl Acad Sci U S A.

[6] Escudero I, Johnstone M (2014). Genetics of schizophrenia. Curr Psychiatry Rep.

[7] Conelea CA, Walther MR, Freeman JB, Garcia AM, Sapyta J, Khanna M, Franklin M (2014). Tic-related obsessive-compulsive disorder (OCD): phenomenology and treatment outcome in the Pediatric OCD Treatment Study II. J Am Acad Child Adolesc Psychiatry.

[8] Gadow KD, Roohi J, DeVincent CJ, Hatchwell E (2008). Association of ADHD, tics, and anxiety with dopamine transporter (DAT1) genotype in autism spectrum disorder. J Child Psychol Psychiatry.

[9] Deik A, Saunders-Pullman R, Luciano MS (2012). Substance of abuse and movement disorders: complex interactions and comorbidities. Curr Drug Abuse Rev.

[10] Asenjo Lobos C, Komossa K, Rummel-Kluge C, Hunger H, Schmid F, Schwarz S, Leucht S (2010). Clozapine versus other atypical antipsychotics for schizophrenia. Cochrane Database Syst Rev.

[11] Lindenmayer JP, Da Silva D, Buendia A, Zylberman I, Vital-Herne M (1995). Tic-like syndrome after treatment with clozapine. Am J Psychiatry.

